# Synovial fluid pharmacokinetics of tulathromycin, gamithromycin and florfenicol after a single subcutaneous dose in cattle

**DOI:** 10.1186/s12917-015-0346-4

**Published:** 2015-02-07

**Authors:** Meredyth L Jones, Kevin E Washburn, Virginia R Fajt, Somchai Rice, Johann F Coetzee

**Affiliations:** Large Animal Clinical Sciences, Texas A&M University College of Veterinary Medicine & Biomedical Sciences, College Station, TX 77843 USA; Veterinary Physiology and Pharmacology, Texas A&M University College of Veterinary Medicine & Biomedical Sciences, College Station, TX 77843 USA; Pharmacology Analytical Support Team (PhAST), Veterinary Diagnostic Laboratory, Iowa State University College of Veterinary Medicine, Ames, IA 50011 USA; Veterinary Diagnostic and Production Animal Medicine, Iowa State University College of Veterinary Medicine, Ames, IA 50011 USA

**Keywords:** Synovial fluid, Tulathromycin, Gamithromycin, Florfenicol, Pharmacokinetics, Bovine

## Abstract

**Background:**

Deep digital septic conditions represent some of the most refractory causes of severe lameness in cattle. The objective of this study was to determine the distribution of tulathromycin, gamithromycin and florfenicol into the synovial fluid of the metatarsophalangeal (MTP) joint of cattle after single subcutaneous administration of drug to evaluate the potential usefulness of these single-dose, long-acting antimicrobials for treating bacterial infections of the joints in cattle.

**Results:**

Twelve cross-bred beef cows were randomly assigned to one of the drugs. Following subcutaneous administration, arthrocentesis of the left metatarsophalangeal joint was performed at various time points up to 240 hours post-injection, and samples were analyzed for drug concentration. In synovial fluid, florfenicol pharmacokinetic parameters estimates were: mean T_max_ 7 +/− 2 hours, mean t_½_ 64.9 +/− 20.1 hours and mean AUC_0-inf_ 154.0 +/− 26.2 ug*h/mL. Gamithromycin synovial fluid pharmacokinetic parameters estimates were: mean T_max_ 8 hours, mean t_½_ 77.9 +/− 30.0 hours, and AUC_0-inf_ 6.5 +/− 2.9 ug*h/mL. Tulathromycin pharmacokinetic parameters estimates in synovial fluid were: T_max_ 19 +/− 10 hours, t_½_ 109 +/− 53.9 hours, and AUC_0-inf_ 57.6 +/− 28.2 ug h/mL.

**Conclusions:**

In conclusion, synovial fluid concentrations of all three antimicrobials were higher for a longer duration than that of previously reported plasma values. Although clinical data are needed to confirm microbiological efficacy, florfenicol achieved a synovial fluid concentration greater than the MIC_90_ for *F. necrophorum* for at least 6 days.

## Background

Lameness is an important cause of production loss and culling in all classes of cattle, making it a significant economic and welfare concern. In a survey of beef cattle operations in the US, 31.6% of herds with greater than 200 cows reported selling cattle due to lameness [1], while 8.4% of death losses of breeding cattle were due to lameness or injury [2], making it the second most common single identifiable cause of death. Deep digital septic conditions represent some of the most refractory conditions causing severe lameness. In a recent report, septic arthritis, tenosynovitis and pedal osteitis represented 15.2% of all beef cattle lameness cases presented to a veterinary teaching hospital [3].

Medical therapy alone is rarely associated with successful resolution of deep septic conditions of the digit, and medical therapy is more often used in combination with surgical approaches, including digit amputation or joint resection. Even when medical therapy is contemplated, there are currently no antimicrobials approved for the treatment of deep limb sepsis in cattle in the United States. When used for deep digital sepsis, antimicrobial drugs are administered by various routes, including systemic, regional intravenous (IV), and intra-articular. Perceived concerns over the ability to achieve sufficient concentrations of drug in the synovial structures by systemic administration has resulted in increased use of local techniques, designed to provide high concentration of drug at the site of infection to increase efficacy. Antimicrobials that exhibit concentration-dependent bacterial killing are most suited for this type of therapy, as these techniques allow for injection of high concentrations of the agent at the site of infection. However, prohibitions against extralabel use in the U.S. or voluntary industry bans preclude the use of the concentration-dependent aminoglycosides, fluoroquinolones and metronidazole in cattle. Regional IV perfusion of ceftiofur [4], tetracycline [5], cefazolin [6] and florfenicol [7] has been evaluated in cattle, but these drugs are considered to depend on time > Minimum Inhibitory Concentration (MIC) to maximize efficacy, which means frequent treatment. However, regional IV perfusion in cattle requires chute restraint, placement of a tourniquet, and daily administration of an antimicrobial drug intravenously in the distal limb. In addition, the cephalosporins may not be administered via an extralabel route in cattle in the U.S, precluding regional IV perfusion.

We propose that drugs with a high volume of distribution should achieve sufficient intra-synovial concentrations by a more easily accomplished route of administration and may produce therapeutic concentrations within joints. Additionally, use of long-acting antimicrobial formulations (that is, longer than 24 hours) would limit the frequency of animal handling and potential disturbance of a surgical site. Tulathromycin, gamithromycin and florfenicol have high volumes of distribution in cattle [8-10] and are available as long-acting formulations. They are expected to demonstrate a time-dependent mode of action, for which the time > MIC for a given pathogen correlates with efficacy, although the pharmacodynamic parameter for any long-acting formulation has not been established. Their spectrum of activity includes Gram-negative anaerobes common in digital disease [11], namely *Fusobacterium necrophorum,* as well as mycoplasmas that have been implicated in arthritis, making these reasonable choices for an investigation to determine whether systemic antimicrobials for deep limb sepsis and septic arthritis of the distal limb might be possible. Finally, synovial fluid concentrations have not been previously assessed with these drugs.

The objective of this study was to determine the distribution of tulathromycin, gamithromycin and florfenicol into the synovial fluid of the metatarsophalangeal (MTP) joint of cattle after a single subcutaneous dose of drug to evaluate the potential usefulness of these drugs for bacterial infections of the joint in cattle. We hypothesized that tulathromycin, gamithromycin and florfenicol would reach detectable, therapeutic and sustained concentrations in synovial fluid following a single parenteral dose.

## Methods

### Animals

Twelve cross-bred beef cows with ages ranging from 2 to 12 years (mean: 6.4 years) were selected for inclusion in this study based on apparent health upon evaluation and no evidence of lameness. Cows were 13–83 days postpartum (mean: 47.2 days), were lactating, and weighed between 480.5 kg and 823.6 kg (mean: 622 kg). Body condition scores of cows ranged from 3.5-7/9 (mean: 4.8/9) [12].

### Experimental design

Four days prior to the start of each of 4 treatment periods, three of the 12 cows were selected if they had already calved (non-random selection). At the study site, they were housed in a paddock and fed *ad libitum* coastal Bermuda grass hay and 2 kg of 20% protein range cubes per cow twice daily. The 3 cows in each treatment period were randomly assigned to a treatment group (tulathromycin, gamithromycin or florfenicol), with the result that four cows were administered each of the three drugs over the course of the study.

On the first day for each treatment period (3 cows/period), cows were weighed on a platform floor scale. Each cow was then restrained in a hydraulic chute with overturning capability. Cows were then overturned and foot restraints applied. The region of the lateral aspect of the metatarsophalangeal (MTP) joint of the left limb was clipped and surgically prepared. Arthrocentesis was performed using a 20ga, 3.8 cm needle and 1.0 mL synovial fluid removed (Time 0). A portion of this sample was evaluated on a refractometer for total protein concentration, and the remainder was placed in a storage tube (Falcon, Tewksbury, MA, USA). A light bandage was placed over the MTP joint, and the cows were returned to standing. Synovial fluid samples were stored at −80°C. Each cow was then administered the assigned antimicrobial subcutaneously (SC) in the neck (tulathromycin 2.5 mg/kg (Draxxin, Pfizer, New York, NY, USA), gamithromycin 6 mg/kg (Zactran, Merial, Duluth, GA, USA), or florfenicol 40 mg/kg (Nuflor Gold, Intervet, Summit, NJ, USA)). If the total dose exceeded 10 mL, multiple injection sites were used. Additional 0.5 mL synovial fluid samples were collected from the MTP joint at 4, 8, 24, 48, 72, 168 and 240 hours post injection, had total protein concentration determined, and were stored at −80°C until completion of the study for determination of drug concentration. Cattle were monitored twice daily for demeanor, appetite, lameness score (1-5/5; 1 – normal, 5 – severely lame) [13], and swelling or drainage from the arthrocentesis site. Each three-cow treatment cycle was repeated four times, resulting in 4 cows receiving each treatment drug. Individual records were maintained for each cow to ensure post-treatment meat withdrawal times were followed.

Procedures used in this study were approved by the Texas A&M University Institutional Animal Care and Use Committee.

### Laboratory analysis

Stored synovial fluid samples were analyzed at the Iowa State University Pharmacology Analytical Support Team (PhAST). Synovial fluid concentrations of florfenicol, gamithromycin and tulathromycin were measured with high-pressure liquid chromatography–tandem mass spectrometry utilizing a LTQ ion trap mass spectrometer (Thermo Scientific, San Jose, CA, USA) coupled to an Agilent 1100 series pump and Autosampler (Agilent Technologies, Santa Clara, CA, USA). Synovial fluid samples or synovial fluid standards were prepared as follows: Briefly, frozen samples or standards were thawed at room temperature. A 200 μL synovial fluid sample was diluted with 0.5 mL of ultrapure water and 0.5 mL of ammonium acetate buffer, pH 4.5. 100 ng and 50 ng of thiamphenicol and roxithromycin, respectively, were added to each tube as internal standards and vigorously mixed by vortex. The samples were centrifuged at 2000 rpm for 20 minutes to pellet solids. The entire diluted supernatant was applied to a solid phase extraction (SPE) cartridge, Strata X-C 33 Polymeric Strong Cation (100 mg/3 mL, Phenomenex, Torrance, CA, USA) which was preconditioned prior with methanol (1 mL), equilibrated with water (1 mL), followed by ammonium acetate buffer, pH 4.5 (1 mL) utilizing gravity for filtration. The sample was subsequently washed with ultrapure water (1 mL), followed by 5% methanol in water (v/v) (1 mL). The SPE cartridges were dried under flow of nitrogen for 5 minutes. Florfenicol (and thiamphenicol) was eluted with 2 fractions of 1 mL portions of 70:30 acetonitrile: methanol into a glass test tube. The SPE cartridges were dried a second time under flow of nitrogen for 5 minutes. Macrolides were eluted from the same column with 2 fractions of a 1 mL 5% ammonium hydroxide in 70:30 acetonitrile: methanol and collected in the same tube as previously described. Samples were evaporated to dryness at 48°C under a stream of nitrogen, reconstituted with 100 μL 25% (v/v) acetonitrile in water. An additional 50 μL of ultrapure water was added, and the entire 150 μL sample was transferred into an injection vial for LC-MS/MS analysis with the injection volume set to 20 μL. The mobile phases consisted of A: 0.1% formic acid in water and B: 0.1% formic acid in acetonitrile at a flow rate of 0.27 mL/min. The mobile phase began at 10% B with a linear gradient to 95% B at 7 minutes, which was maintained for 2 minutes, followed by re-equilibration to 10% B. Separation was achieved with an ACE3 C18 column (150 mm × 2.1 mm, 3 μm particles, Advanced Chromatography Technologies, LTD (MacMod, Chadds Ford, PA, USA) maintained at 40°C.

Florfenicol eluted at 6.03 minutes, gamithromycin eluted at 5.64 minutes, tulathromycin eluted at 4.79 minutes, roxithromycin eluted at 6.75 minutes, and thiamphenicol eluted at 5.10 minutes. Three SRM transitions were monitored for all target analytes. The quantifying ions for florfenicol were 218.89, 335.94, and 357.25 m/z. The quantifying ions for gamithromycin were 462.44, 601.50, and 619.47 m/z. The quantifying ions for tulathromycin were 230.96, 251.14, and 289.36 m/z. The quantifying ions for internal standards roxithromycin and thiamphenicol were 522.34, 558.22, and 679.35 m/z and 227.10, 290.05, and 282.00 m/z, respectively. Synovial fluid concentration of target analytes in unknown samples were calculated by the Xcalibur software based on the calibration curve. Results were then viewed in the Quan Browser portion of the Xcalibur software. The standard curve determined using bovine synovial fluid ranged 1 to 1000 ng/mL for florfenicol and 1 to 5000 ng/mL for the macrolides and was accepted when the correlation coefficient exceeded 0.99 and measured values were within 15% of the actual values. Sample concentrations not bracketed by range of the standard curve were repeated using a lesser volume of synovial fluid and final concentrations were back-calculated using the appropriate dilution factor dependent on the volume of synovial fluid analyzed. The inter-assay CV for mid and high range controls (100 ng/mL and 500 ng/mL from the calibration curve) were 3.1 for florfenicol, 5.7 for gamithromycin, and 4.3 for tulathromycin. There was insufficient volume of unknown samples for duplicate runs, and intra-assay CV was not determined.

Noncompartmental analysis was performed using industry standard software (WinNonLin 6.3, Pharsight) to estimate the pharmacokinetic parameters in synovial fluid for each individual animal. The following parameters were estimated for each animal: time of peak serum drug concentration (T_max_), peak drug concentration (C_max_), apparent elimination half-life (t_1/2_, calculated as ln(2)/λ_z_, λ_z_ being the first order rate constant associated with the terminal portion of the time-concentration curve as estimated by linear regression of time vs. log concentration), area under the time-concentration curve from time zero to the last observed concentration (AUC_0-obs_, calculated by the linear trapezoidal rule), area under the time-concentration curve from time zero extrapolated to infinity (AUC_0-inf_, calculated by adding the last observed concentration divided by λ_z_ to the AUC_0-obs_), area under the moment curve from time zero to last observed concentration (AUMC_0-obs_), area under the moment curve from time zero extrapolated to infinity (AUMC_0-inf_), mean resident time estimated using time zero to last observed concentrations (MRT_0-obs_, calculated as AUMC_0-obs_/AUC_0-obs_), and mean residence time estimated using time zero to infinity (MRT_0-inf_, calculated as AUMC_0-inf_/AUC_0-inf_). Mean parameters for each drug were then calculated from individual animal estimates.

## Results

**Figure 1:**
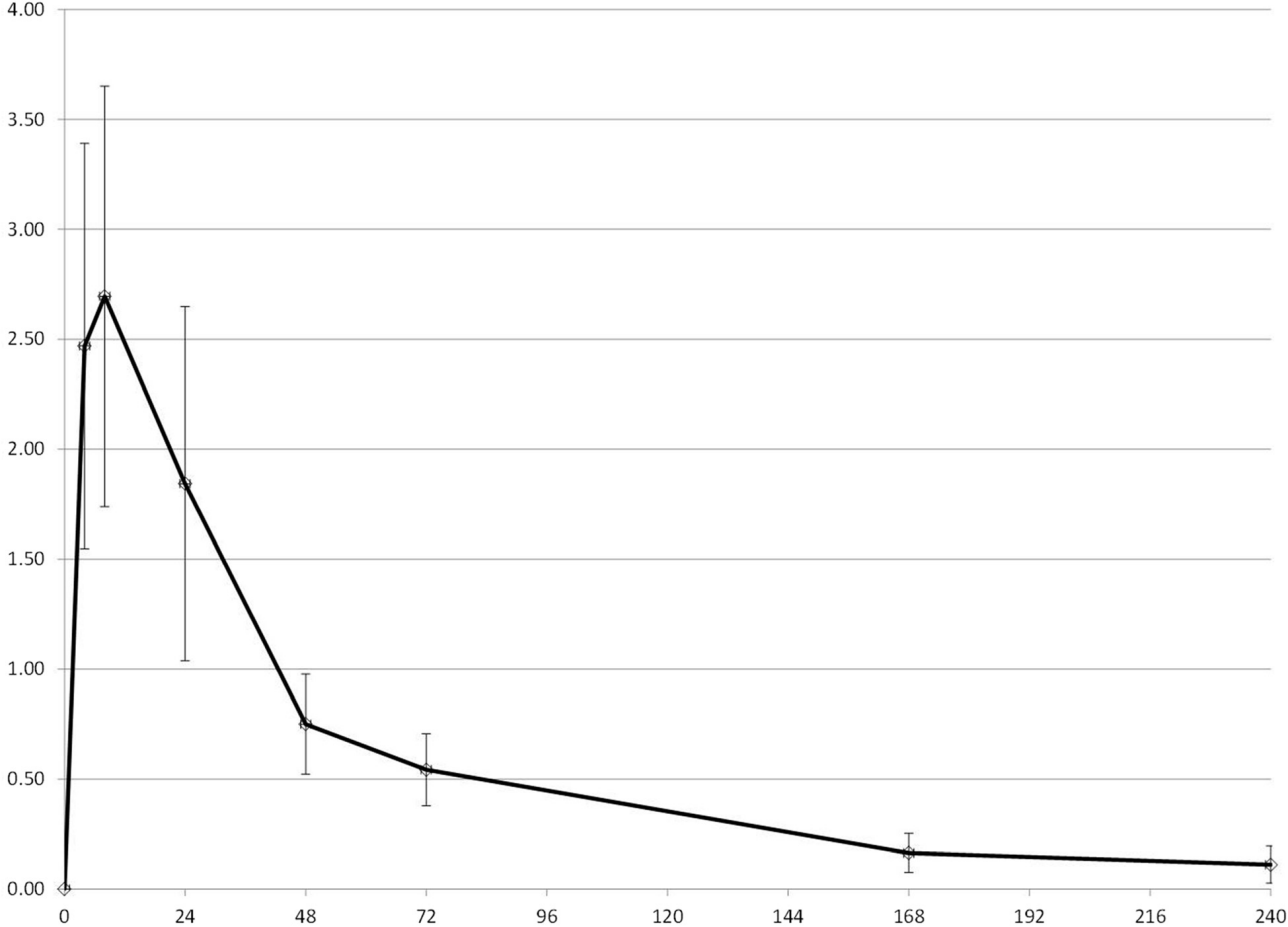
**Florfenicol concentration in synovial fluid.** Mean synovial fluid concentrations of florfenicol (mcg/mL) after single SC injection of 40 mg/kg in 4 adult beef cows. The x-axis represents hours post administration.

**Figure 2:**
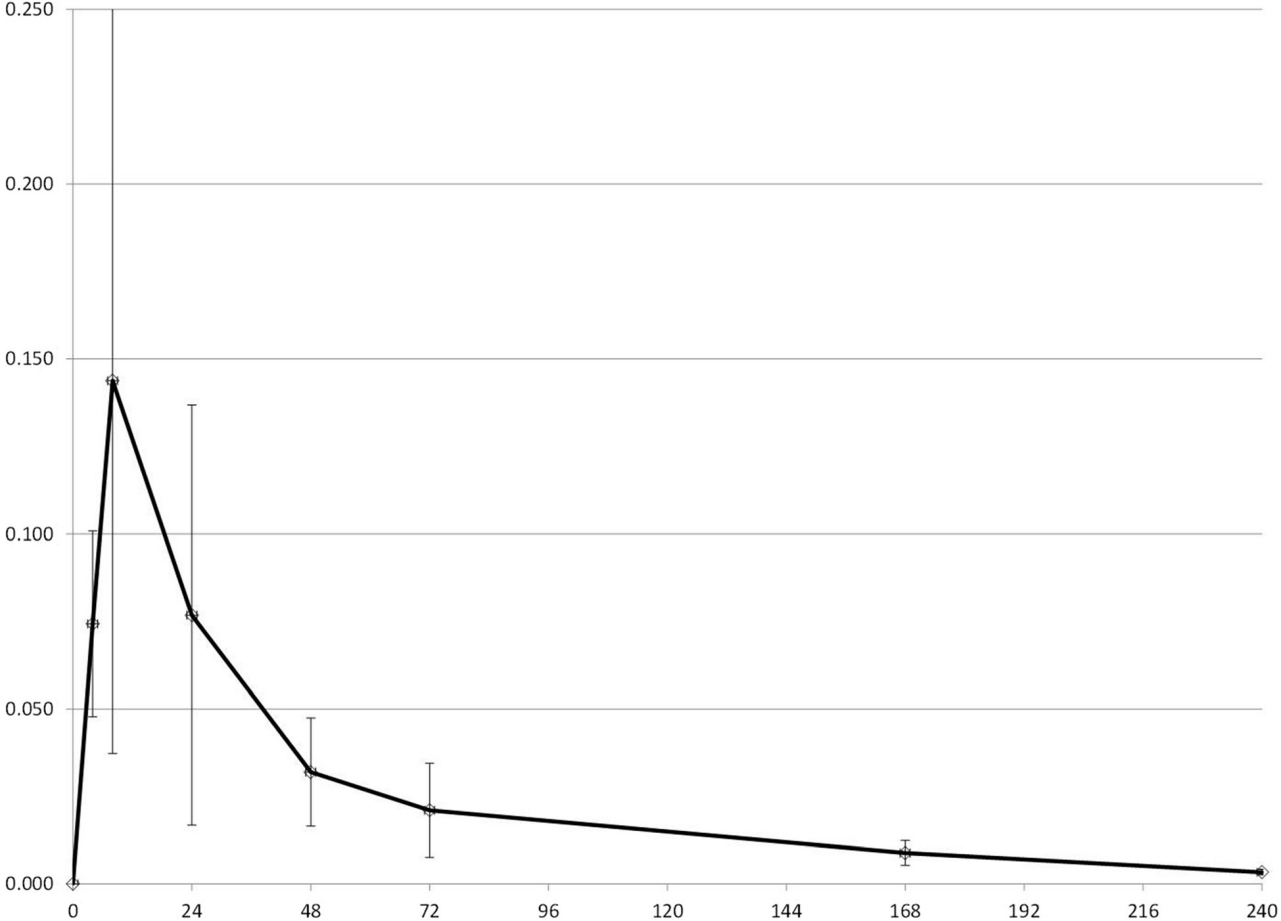
**Gamithromycin concentration in synovial fluid.** Mean synovial fluid concentrations of gamithromycin (mcg/mL) after single SC injection of 6 mg/kg in 4 adult beef cows. The x-axis represents hours post administration.

**Figure 3:**
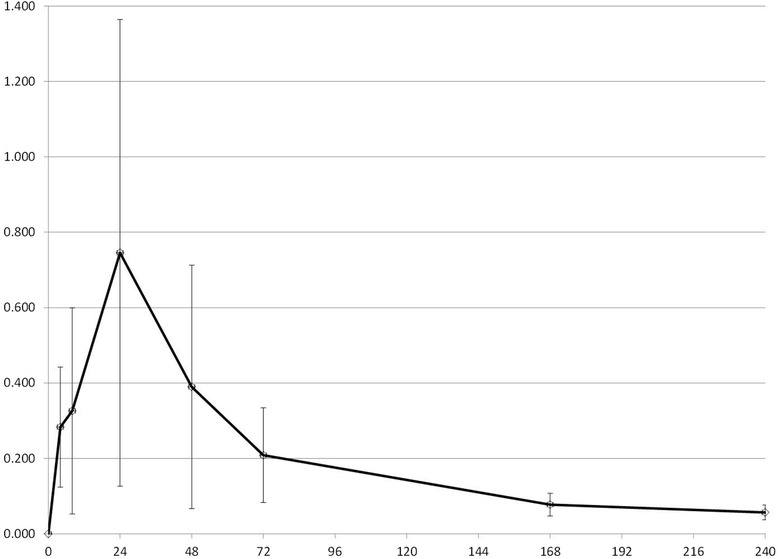
**Tulathromycin concentration in synovial fluid.** Mean synovial fluid concentrations of tulathromycin (mcg/mL) after single SC injection of 2.5 mg/kg in 4 adult beef cows. The x-axis represents hours post administration.

All synovial fluid samples were successfully collected as scheduled (see Figures 1, 2 and 3 for synovial fluid concentrations). Cows remained apparently healthy throughout the study as indicated by normal attitude and appetite. No cow was scored with a lameness score greater than 1 (normal) at any point in the study. Mild swelling was noted in some cows at the site of arthrocentesis; this swelling never interfered with sampling nor was painful to pressure or produced drainage. Total protein levels were measured on all synovial fluid samples and ranged from 0.1-1.6 g/dL (mean: 0.52 g/dL).

Mean synovial fluid pharmacokinetic parameters for florfenicol, gamithromycin and tulathromycin were calculated (Table 1).

**Table 1:** **Synovial fluid pharmacokinetic parameters**

		**Florfenicol**		**Gamithromycin**		**Tulathromycin**	
		**Mean**	**SD**	**Mean**	**SD**	**Mean**	**SD**
**C**_**max**_	mcg/mL	2.71	0.93	0.14	0.11	0.79	0.57
**T**_**max**_	hr	7	2	8	0	19	10
**λz**	/hr	0.0115	0.0035	0.0099	0.0038	0.0077	0.0038
**t**_**½λz**_	hr	64.9	20.1	77.9	30.0	109.0	53.9
**AUC**_**last**_	mcg*hr/mL	142.0	17.5	6.2	2.8	49.7	29.1
**AUC**_**0-inf**_	mcg*hr/mL	154.0	26.2	6.5	2.9	57.6	28.2
**AUC % Extrap**		7.1	6.3	6.2	2.8	18.1	12.1
**AUMC**_**0-obs**_	mcg*hr^2^/mL	7623	2577	331	122	3323	1577
**AUMC**_**0-inf**_	mcg*hr^2^/mL	11860	7054	469	124	6490	1311
**MRT**_**0-obs**_	hr	53.5	15.4	55.9	14.2	71.7	14.4
**MRT**_**0-inf**_	hr	74.1	32.9	75.2	12.3	136.5	65.0

T_max_: time of peak serum drug concentration; C_max:_ peak drug concentration; t_1/2_: apparent elimination half-life calculated as ln(2)/λ_z_, λ_z_ being the first order rate constant associated with the terminal portion of the time-concentration curve; AUC_0-obs_: area under the time-concentration curve from time zero to the last observed concentration; AUC_0-inf_: area under the time-concentration curve from time zero extrapolated to infinity; AUMC_0-obs_: area under the moment curve from time zero to the last observed concentration; AUMC_0-inf_: area under the moment curve from time zero extrapolated to infinity; MRT_0-obs_: Mean Resident Time calculated as AUMC_0-obs_ /AUC_0-obs_; MRT_0-inf_: Mean Residence Time calculated as AUMC_0-inf_ /AUC_0-inf._

Mean pharmacokinetic parameters in synovial fluid estimated via noncompartmental analysis after SC administration of florfenicol (40 mg/kg), gamithromycin (6 mg/kg), and tulathromycin (2.5 mg/kg) (n = 4/group).

## Discussion

The objective of our study was to demonstrate that potentially effective concentrations of 3 antimicrobial drugs are achieved in synovial fluid after subcutaneous administration of a single dose of long-acting time-dependent drugs, so that logistically challenging methods of drug administration such as regional IV infusion can be avoided. Our sampling strategy focused on determining concentrations in the elimination phase of drug disposition, because we were mainly interested in demonstrating overall drug exposure and elimination rate to compare with previously described plasma disposition of the drugs [9,14-16]. Characterizing the rate of distribution of drugs into the joint space was a low priority, so we purposely did not collect many samples in the early hours after drug administration but did sample up to 240 hours. We chose not to evaluate comparative plasma concentrations in the present study due to financial constraints, and because published data are available. This sampling strategy therefore might have resulted in inaccurate estimates of C_max_. Published mean plasma C_max_ estimate for florfenicol was 5.9 mcg/mL [17], approximately twice our estimate of 2.7 mcg/ml. For gamithromycin, C_max_ has been estimated to be 0.75 mcg/mL [9], significantly higher than our estimate of 0.14 mcg/mL. And the C_max_ estimate for tulathromycin was 0.28 mcg/mL [16] in one study, which is lower than our estimate of 0.79 mcg/ml. However, because we had a limited number of samples in the time range of the reported plasma T_max_ of all 3 drugs (5 hrs for florfenicol [17], 8 hrs for gamithromycin [9], 3 hrs for tulathromycin [16]), and because we expect drug distribution into the joint space to not be immediate, these differences are probably of little significance clinically.

More important are the comparisons between plasma t_1/2_ and synovial t_1/2_ as well as between the time synovial concentrations remain above MIC of target organisms as compared to plasma concentrations. We assumed that the appropriate pharmacodynamic parameter was time > MIC, but pharmacodynamic parameters for long-acting formulations or for macrolides have not been well characterized in the literature. T_1/2_ of florfenicol in synovial fluid was estimated to be approximately 65 hrs, compared to reported plasma t_1/2_ of 38 hrs [17]. T_1/2_ of gamithromycin in synovial fluid was estimated to be 78 hrs vs. 51 hrs in plasma as previously reported [9]. Finally, t_1/2_ of tulathromycin in synovial fluid was estimated in the present study to be 109 hrs, as compared to the published plasma t_1/2_ of 64 hrs [16]. Therefore, it appears that drug elimination from synovial fluid is slower than from plasma due to delayed presentation to the organs of elimination, a potential advantage for medical therapy.

Equally important to compare is the amount of time that synovial fluid concentrations remained above a particular concentration, since this is likely to be predictive of efficacy. Recognizing that a direct comparison between pathogen MIC and synovial fluid concentration is not defensible in the absence of supportive clinical data, a qualitative comparison is helpful in suggesting the potential for efficacy. Synovial concentrations of florfenicol in the present study remained above 0.5 mcg/mL for approximately 72 hours, which is similar to the previously reported plasma concentrations. Synovial concentrations of gamithromycin in the present study compared favorably with previously reported plasma concentrations: synovial concentrations were 0.02 mcg/mL at 72 hrs, as compared to 0.026 mcg/mL previously reported. Finally, synovial concentration of tulathromycin in the present study averaged 0.078 mcg/mL at 168 hrs, whereas previously reported estimates (from visual examination of graphical data) were between 0.01 and 0.02 mcg/mL.

MICs for several target pathogens for these 3 drugs have been reported: Synovial fluid concentrations of florfenicol in the present study would be expected to be above *Fusobacterium necrophorum* and *Bacteroides melaninogenicus* MIC (MIC_90_: 0.25 mcg/mL) for at least 6 days, but not above reported MICs for *Trueperella pyogenes* (MIC_90_: 16 mcg/mL [18]), although these were uterine isolates. Susceptibility data related to gamithromycin and Gram-negative anaerobic isolates likely to be responsible for digital sepsis are not available, so predictions cannot be made for this drug. Synovial fluid concentrations of tulathromycin do not appear to remain above the reported MIC_50_ of *Fusobacterium necrophorum* (2 mcg/mL); however, tulathromycin is approved for use in the treatment of footrot, and the plasma concentrations also do not reach these concentrations, so the PK/PD relationship is likely more complicated than a simple comparison of concentration to MIC. One important caveat to these comparisons of pharmacokinetic values to published data on florfenicol and tulathromycin is that there have been two commercially available florfenicol preparations (Nuflor, Schering Plough, Summit, NJ, USA; Nuflor Gold, Intervet, Roseland, NJ, USA) , and one study of tulathromycin was performed using prototype formulations [15].

A question might arise about the impact of sample collection on inflammation in the joint and its effects on drug concentrations. A previous study [7] used indwelling catheters to facilitate sample collection to evaluate synovial fluid pharmacokinetics after regional perfusion in the metatarsophalangeal joint. Synovial fluid samples for this project, however, were readily obtained with needle arthrocentesis at each time point. This technique potentially minimized inflammation in the joint by eliminating the use of an indwelling foreign body for 240 hours. While total protein concentration of synovial fluid as measured on a refractometer is an imperfect indicator of inflammation, the values obtained at each time point fell well below medial total protein levels seen in cattle with various forms of noninfectious joint disease [19]. This, combined with no clinical evidence of inflammation (swelling, drainage, lameness) in any study joint, provides an indication that this procedure is both effective at obtaining sufficient sample for drug analysis and minimizing inflammatory response. Systemic administration of antimicrobial drugs may also help prevent septicemia of the digital vasculature, which has been reported after regional IV perfusion of lidocaine when digital sepsis was present [20].

No products used in this study are approved for use in lactating dairy cattle and their use may only be advocated in beef cattle and non-lactating dairy cattle. Products including ampicillin, ceftiofur and oxytetracycline make more appropriate choices for dairy cattle, but need to be evaluated for their synovial fluid pharmacokinetics in adult cattle. Oxytetracycline concentrations in synovial fluid have been evaluated after IM injection in healthy calves [21,22], and IV administration in calves with experimentally-induced synovitis [23], but these drugs were not included in the present study.

## Conclusions

In conclusion, synovial fluid concentrations of all three drugs were higher for a longer duration than that of previously reported plasma values. Although florfenicol achieved a synovial fluid concentration greater than the MIC_90_ for *F. necrophorum* for at least 6 days, clinical data are needed to confirm microbiological efficacy.

## Abbreviations

AUC: Area under the curve
AUMC: Area under the moment curve
Cmax: Peak drug concentration
MIC: Minimum inhibitory concentration
MRT: Mean residence time
MTP: Metatarsophalangeal
t1/2: Elimination half-life
Tmax: Time of peak drug concentration
